# Utilization of somatic fusion techniques for the development of HLB tolerant breeding resources employing the Australian finger lime (*Citrus australasica*)

**DOI:** 10.1371/journal.pone.0255842

**Published:** 2021-08-10

**Authors:** Manjul Dutt, Lamiaa M. Mahmoud, Karen Chamusco, Daniel Stanton, Christine D. Chase, Ethan Nielsen, Maria Quirico, Qibin Yu, Frederick G. Gmitter, Jude W. Grosser

**Affiliations:** 1 Citrus Research and Education Center, University of Florida, Lake Alfred, FL, United States of America; 2 Faculty of Agriculture, Pomology Department, Mansoura University, Mansoura, Egypt; 3 Horticultural Sciences Department, University of Florida, Gainesville, FL, United States of America; Fujian Agriculture and Forestry University, CHINA

## Abstract

The Australian finger lime is a unique citrus species that has gained importance due to its unique fruit characteristics and perceived tolerance to Huanglongbing (HLB), an often-fatal disease of citrus trees. In this study, we developed allotetraploid finger lime hybrids and cybrids by utilizing somatic cell fusion techniques to fuse diploid ‘OLL8’ sweet orange or ‘Page’ tangelo callus-derived protoplasts with finger lime (FL) mesophyll-derived protoplasts. Six somatic fusions were regenerated from the ‘OLL8’ + FL fusion, while three putative cybrids were regenerated from the ‘Page’ + FL fusion. Ploidy levels and nuclear-expressed sequence tag derived simple sequence repeat (EST-SSR) markers confirmed the somatic hybrid production, and mitochondrial DNA primer sets confirmed the cybrid nature. Several trees produced by the somatic fusion remained HLB negative even after 6 years of growth in an HLB-endemic environment. Pathogenesis related (PR) and other genes that are often upregulated in HLB-tolerant trees were also upregulated in our somatic fusions. These newly developed somatic fusions and cybrids could potentially be used as breeding parents to develop the next generation of improved HLB-tolerant rootstocks and scions.

## Introduction

Finger lime (*Citrus australasica* F. Muell) is an Australian native citrus species. Finger lime trees likely originated in South Asia and migrated to Australia during the early Pliocene epoch, where they further developed into the modern-day finger lime. This species is characterized by its round to teardrop-shaped juice vesicles that burst into individual juice sacs when the fruit is cut. The finger-shaped fruit is sold for its juice vesicles, which can be separated into individual "pearls" resembling caviar, giving it the nickname citrus caviar [1], whereas juice vesicles of most citrus cultivars tend to adhere together [2]. Recently, finger limes have displayed tolerance to Huanglongbing (HLB), a major disease in citrus caused by the phloem-limited bacterium *Candidatus* Liberibacter asiaticus (*Ca*Las) [3]. Earlier studies have demonstrated that susceptibility and tolerance responses towards HLB depend on the specific citrus host and this trait can be utilized for the development of HLB tolerant citrus [3,4].

Several wild citrus species have evolved to coexist with hostile pathogens such as *Ca*Las and can thrive under an HLB endemic environment [4] In general the most effective and sustainable approach to disease control is the introgression of resistance genes from HLB-tolerant species into a susceptible cultivar [5,6]. Although most commercial citrus cultivars can be infected by *Ca*Las and often succumb to HLB, several of the *Ca*Las-tolerant wild and cultivated species are being used to develop HLB-resistant citrus cultivars [3,7,8]. Of late, breeding to introgress HLB resistance into cultivated cultivars has become a staple approach in many citrus breeding programs [9,10]. Mainly diploid cultivars are commonly utilized for citrus improvement efforts, but more recently, several tetraploid selections have also been used [11–13].

Tetraploid induction is a beneficial tool for plant breeding and improvement [14,15]. Tetraploid plants can be either autotetraploid or allotetraploid. Autotetraploid plants arise from a natural or chemically induced (colchicine, oryzalin, or trifluralin) doubling of a diploid genome, whereas allotetraploid plants are usually a product of the somatic fusion process and arise from the combination of two different diploid genomes [16–18]. Tetraploid citrus can be utilized directly as improved scion or rootstock cultivars [19,20]; however, they can also be utilized to develop seedless triploid cultivars [21].

The protoplast-mediated somatic fusion process has been a successful and valuable technique used in citrus to produce unique autotetraploid and allotetraploid breeding parents that combine elite diploid selections [21,22]. In some cases, somatic fusion experiments produced diploid plants with morphological features of the presumably non-embryogenic leaf parent, known as cybrids [23]. This approach can generate tremendous genetic diversity in zygotic progeny and is a powerful tool for packaging all necessary disease-resistant traits into horticulturally desirable cultivars [21,24,25]. In the present study, we used a somatic fusion technique to develop novel allotetraploid and cybrid somatic fusion plants between the Australian finger lime and selected sweet orange and tangelo cultivars to develop elite HLB-tolerant tetraploid and cybrids.

## Materials and methods

### Protoplast isolation, PEG fusion, protoplast culture, and plant regeneration

Embryogenic calli of ‘OLL8’ sweet orange and ‘Page’ tangelo were initiated from undeveloped ovules and cultured on DOG medium, according to Grosser and Gmitter [21]. Proliferated friable calli obtained from these ovules were sub-cultured every 4 weeks in the same medium. One-year-old callus cells that were actively dividing were used to isolate protoplasts as outlined by Grosser and Gmitter [26]. Fully expanded finger lime leaves were collected from plants in the greenhouse, sterilized in a 5% commercial bleach solution, and cut into thin strips before incubation in the same enzyme solution used to obtain callus protoplasts. Protoplasts were purified on a sucrose/mannitol gradient, and protoplasts were fused using the polyethylene glycol method. Somatic embryo and plant recovery were performed as previously described by Grosser and Gmitter [26].

### Flow cytometer analysis

Ploidy analysis was performed using a CyFlow^®^ Cube 6 flow cytometer (Sysmex America, Inc., Lincolnshire, IL, USA). A small leaf piece (approximately 0.4 cm^2^) was chopped using a sharp blade in nuclei extraction buffer. This mixture was strained through a 45-μm nylon mesh screen and stained with DAPI, a fluorescent nuclear stain, according to the manufacturer’s instructions for the CyStain UV Precise P Automate Kit (Sysmex America, Inc.). The position of the 2N histogram peak was determined using nuclear DNA obtained from the key lime (*Citrus aurantifolia* Swingle) diploid standard.

### Leaf area and stomatal trait analysis

Diploid and tetraploid leaves were collected from mature field trees. The leaf area was measured using an LI-3100C area meter (LICOR, Lincoln, NE, USA) calibrated to 0.01 cm^2^. For stomatal trait analysis, leaf samples were washed using deionized (DI) water and fixed in a 4% paraformaldehyde solution buffered with 1x phosphate-buffered saline (PBS). Samples were dehydrated in an ethanol series (30%, 50%, 70%, 85%, 95%, and 100%). The tissue was incubated in 100% ethanol overnight at 4°C. The tissue was then dried using a Ladd 28000 critical point dryer (Ladd Research Industries, Williston, VT, USA) and mounted on double-sided 12 mm carbon stickers (Electron Microscopy Sciences, Hatfield, PA, USA) on scanning electron microscope (SEM) stubs. Leaf samples were sputter-coated using a Ladd 30800 sputter coater (Ladd Research Industries) with a gold/palladium target. Images of stomata were captured using a Hitachi S4000 SEM (Hitachi, Tokyo, Japan). Twenty randomly captured images were analyzed for the average number of stomata in each group. Stomata from each group were also selected at random for area analysis using ImageJ software at 600X magnification.

### Somatic fusion confirmation using SSR marker analysis

DNA was extracted from approximately 100 mg of fresh leaves using a GeneJET Plant Genomic DNA Purification Mini Kit (Thermo Fisher Scientific, Franklin, MA, USA) following the manufacturer’s protocol. The extracted DNA concentration was measured using a NanoDrop™ 1000 spectrophotometer (Thermo Fisher Scientific) and normalized to 25 ng/μL. Polymerase chain reaction (PCR) amplifications were performed using 10 different SSR primer sets to generate gene-specific amplicons using a T100™ Thermal Cycler (Bio-Rad Laboratories, Inc. Hercules, CA, USA). Fragment separation was performed using an ABI PRISM 3130 xl Genetic Analyzer (Applied Biosystems, Foster City, CA, USA). A universal M13 primer (5′–GTTGTAAAACGACGGCCAGT– 3′) was fluorescently labeled (with either 6-FAM, VIC, NED, or PET) and added as a common tail to the 5′ end of the forward SSR primers (S1 Table). SSR markers were analyzed using GeneMarker 1.40 (SoftGenetics LLC, State College, PA, USA).

### Confirmation of cybrid progeny by organelle genotyping assay

Plastid and mitochondrial genotypes of fusion partners and products were analyzed by PCR amplification of DNA products by conventional PCR and separating amplicons by polyacrylamide gel electrophoresis [27]. The plastid and mitochondrial DNA primer sets used in the present study are listed in S2 Table and outlined in the previous citrus genotyping studies [27,28].

### *Ca*Las diagnostics and gene expression analysis

To diagnose *Ca*Las*-*infected leaves, genomic DNA was isolated from the midveins of young, fully expanded leaf tissues using the GeneJET Plant Genomic DNA Purification Kit (Thermo Fisher Scientific). DNA was normalized to 25 ng/μL before performing qPCR using a StepOnePlus™ Real-Time PCR System (Thermo Fisher Scientific). Detection of *Ca*Las genomic DNA was determined by qPCR using TaqMan™ Gene Expression Master Mix and CQUL primers (S3 Table) to amplify a *Ca*Las rplJ/rplL ribosomal protein gene [29].

Gene-specific primers (S4 Table) were designed using the real-time PCR tool available at www.idtdna.com (Integrated DNA Technologies, Coralville, IA, USA). According to the manufacturer’s protocol, RNA was isolated from approximately 100 mg of leaf tissue using Direct-zol™ RNA Miniprep (Zymo Research, Irvine, CA, USA). RNA concentration was determined using a NanoDrop™ 1000 spectrophotometer (Thermo Fisher Scientific). Single-strand cDNA was produced using a RevertAid First Strand cDNA Synthesis Kit (Thermo Fisher Scientific). The expression of PR1, PR2, 20G-Fe, ABF3, ZIP10, CAM8, EXP-A4, and ABC transporter C genes was analyzed using qPCR. A PowerUp™ SYBR^®^ Green Master Mix (Thermo Fisher Scientific) with 50 ng cDNA and gene-specific primers for each of these genes were used to conduct q-PCR with three replicates for each reaction. OLL8 expression was used as the control, and citrus β-actin was used as a housekeeping gene [30]. Relative gene expression was calculated using the 2^-ΔΔCt^ method [31].

### Statistical analysis

Data were analyzed using a one-way ANOVA statistical test with Tukey’s honestly significant difference post hoc test (*P* ≤ 0.05) using JMP Pro 15 (SAS Institute, Cary, NC, USA).

## Results and discussion

### Somatic fusion resulted in the production of allotetraploids

**Fig 1:**
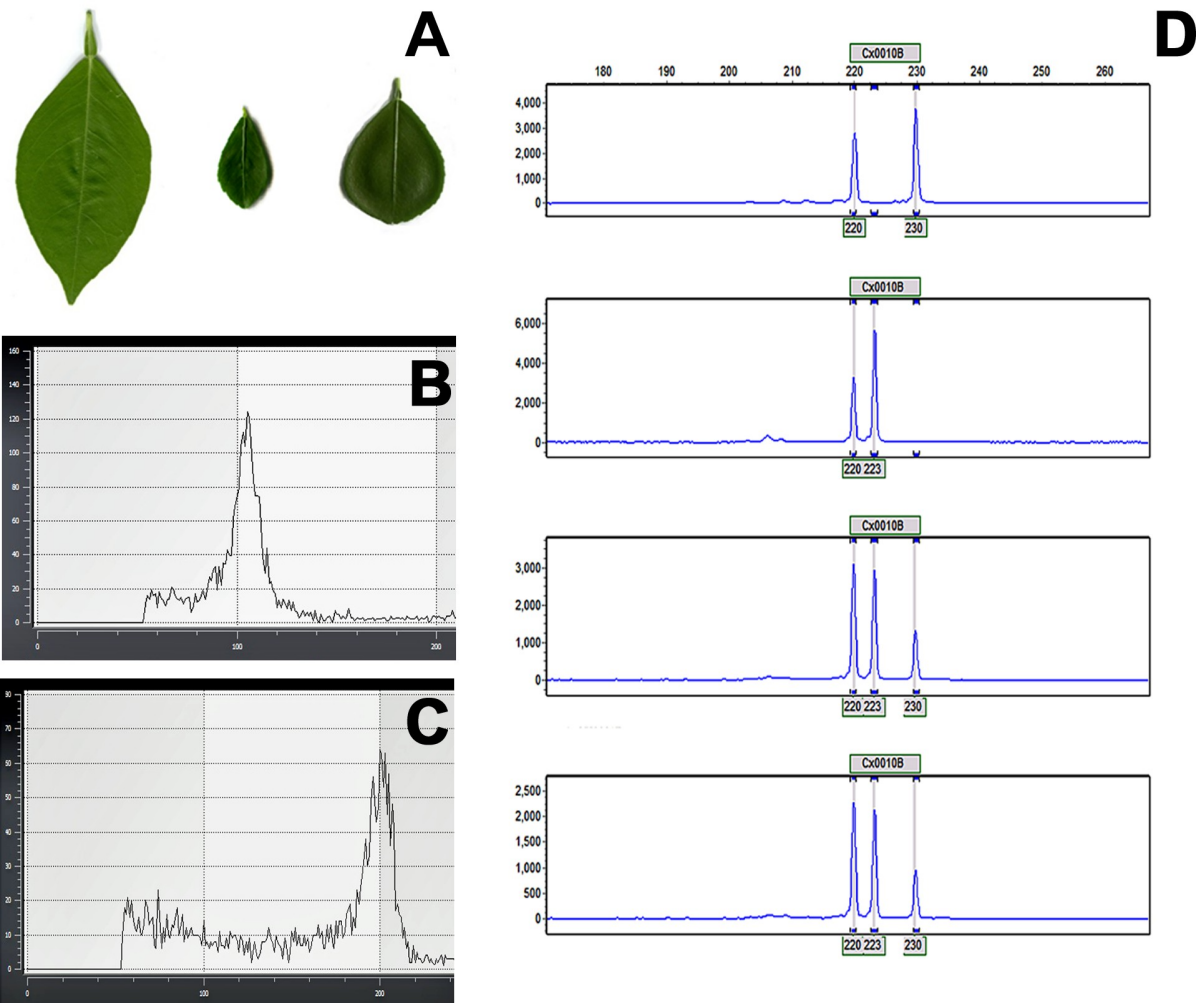
Characterization of somatic fusions. A) Morphological changes in leaves of the diploid parents (left, OLL8; middle; FL) and an OLL8+FL allotetraploid (right). B) and C) Ploidy analysis using flow cytometry. Diploid (B) and tetraploid (C) peaks derived from control and OLL8 + finger lime somatic fusion lines. D) A chromatogram of EST-SSR marker CX0010 labeled with 6-FAM generated from ABI trace files by GeneMarker® software (SoftGenetics). Top panel; OLL8 sweet orange (2X), Middle panel; finger lime (2X) Third and fourth panel; two independent OLL8 + FL (4X) lines.

Finger limes have been reported to be HLB-tolerant [3]. Because finger limes are monoembryonic, variation in HLB tolerance between the seedling-derived populations is expected. In this study, we selected an HLB-tolerant finger lime clone (DPI 50–36) as the leaf parent for all somatic fusion experiments. Numerous potential allotetraploids and cybrids were regenerated following successful fusion with protoplasts of embryogenic ‘OLL8’ sweet orange and ‘Page’ tangelo calli. We selected these two accessions because they are well adapted to Florida’s climate. Sweet oranges are hybrids between pummelo and mandarin [32], and ‘OLL8’ is an improved sweet orange that was recently released from the citrus breeding program of the University of Florida [33]. The ‘Page’ tangelo is a complex hybrid with grapefruit and mandarin genetics and is well adapted in the Florida environment [34]. Six somatic fusions were regenerated from the ‘OLL8’ fusion experiment, whereas ‘Page’ produced three putative cybrids in this study. Somatic fusions could be easily identified by their leaf morphology (Fig 1A), whereas cybrids were identified as finger lime (mesophyll parent protoplast donor) plants regenerated from the somatic fusion experiment. All recovered putative tetraploid and cybrid plantlets were micrografted to vigorous trifoliate-leaved rootstocks to expedite whole plant recovery and growth. All regenerated plants were evaluated by flow cytometry to determine diploid and tetraploid plants based on the representative histogram of the fluorescence nuclear intensities (Fig 1B and 1C). Two of the six tetraploids did not survive in the field and perished within the first year of planting. The remaining 4 were evaluated in this study.

### Tetraploids are morphologically and anatomically different from diploids

The four OLL8 + FL somatic fusions were evaluated in detail. We measured the ‘OLL8’ sweet orange leaf area and the finger lime parent and four selected somatic fusions using an LI-3100C area meter (Table 1). We observed that tetraploid somatic fusion leaves were significantly larger than those of the finger lime parent (Fig 1A). Compared with finger lime leaves, the somatic fusion leaves were on average 2–3-fold larger, ranging from 2.62 to 3.28 cm^2^, whereas the ‘OLL8’ leaves were much larger, being on average 35.86 cm^2^ (Fig 1A). We did not observe any incompatibility issues during the somatic fusion process between the finger lime and the ‘OLL8’ sweet orange used in this study. Allotetraploid intergeneric somatic hybrid plants in prior studies have been produced between citrus and sexually compatible and incompatible relatives [17,35]. To the best of our knowledge, the present study is the first to provide evidence of somatic fusion derived tetraploid plant production and characterization utilizing the finger lime as one of the fusion parents.

**Table 1:** Leaf area measurements of the allotetraploid somatic hybrids between OLL8 sweet orange and the finger lime and the diploid parents.

Cultivar	Leaf area (cm^2^)
OLL8	35.86 ± 9.40^a^
Finger lime	0.90 ± 0.02^c^
OLL8 + finger lime 1	2.62 ± 0.17^b^
OLL8 + finger lime 2	3.08 ± 0.39^b^
OLL8 + finger lime 3	3.18 ± 0.16^b^
OLL8 + finger lime 4	3.28 ± 0.34^b^

* Means separation by Tukey’s honestly significant difference test (P≤ 0.05). Values represent means ± standard error. Each number is an average of 10 replicates.

**Fig 2:**
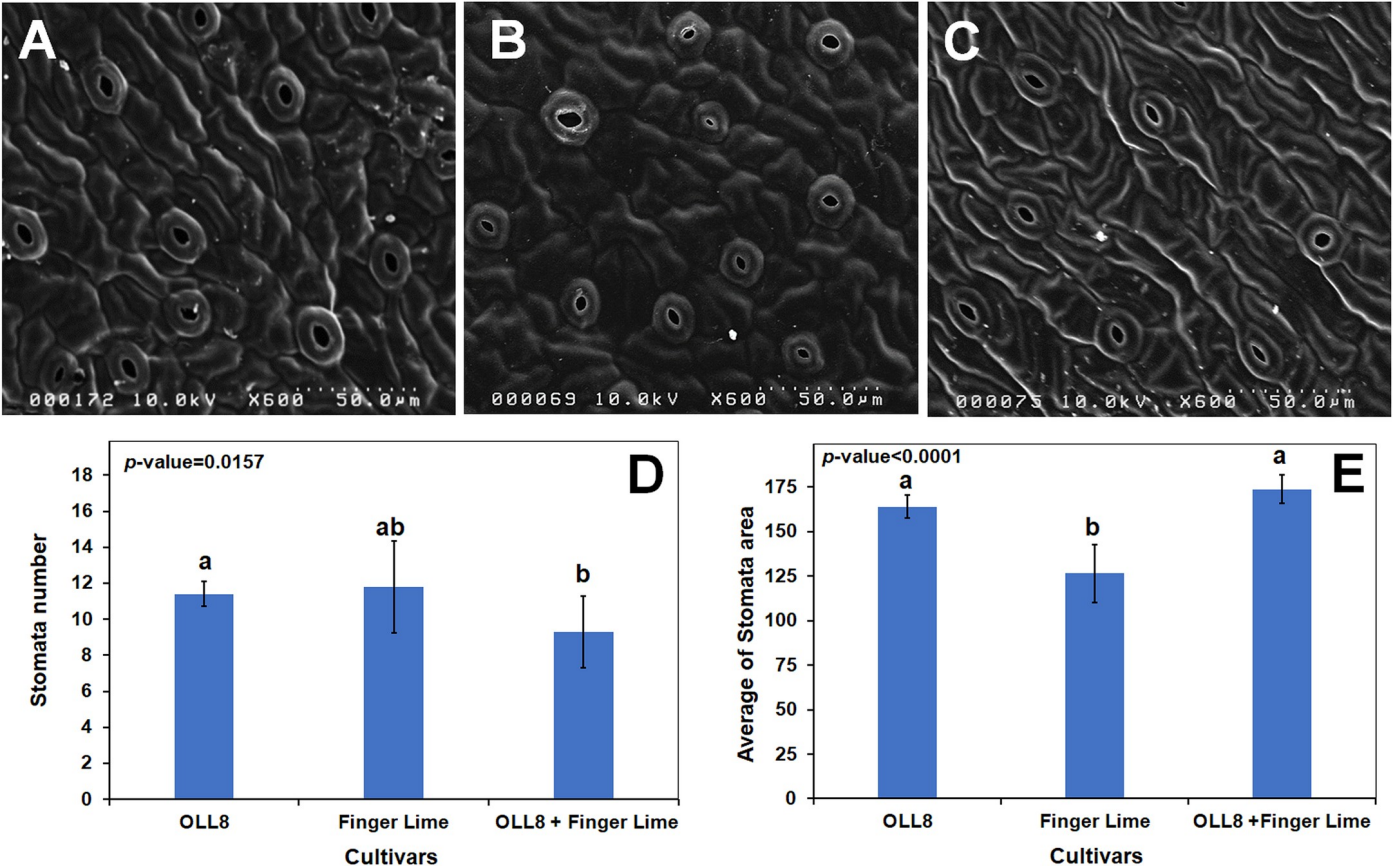
Stomatal trait analysis in diploid and tetraploid finger lime leaves. Top panel (A-C) are Scanning Electron Microscopy (SEM) images demonstrating differences in stomata numbers in A) OLL8, (B) finger lime and a (C) tetraploid somatic fusion. Lower panel are bar graphs comparing stomata number (D) and average stomatal area (E) in diploid parents and the tetraploid somatic fusion.

Stomatal measurements obtained from SEM of diploid and tetraploid leaves revealed that although there was relatively the same number of stomata in diploid and tetraploid leaves when leaf size was considered, there was a decrease in the stomatal number per unit area in the tetraploid leaves (*p*-value = 0.0157). We observed an increase in the average stomatal area on tetraploid leaves (*p*-value ≤ 0.0001) compared with stomata on the diploid finger lime parent but was not significantly different when compared to the ‘OLL8’ callus parent (Fig 2). Tetraploid leaves are usually larger than diploid ones, which concomitantly results in a decrease in stomatal density. Most stomatal comparison studies have focused on comparing diploid cultivars and their autotetraploids [36,37] but in this study we compared the diploid parents with their allotetraploids. Because our allotetraploids have additive genomes [22] as a result of the fusion of two distinct species, it is possible that the lack of statistical difference in the stomatal number between the diploid and tetraploid leaves could be related to the genetic influence of the ‘OLL8’ parent.

### EST-SSR analysis confirmed the production of allotetraploids

To confirm our earlier visual observations and subsequent flow cytometry results, we performed EST-SSR analysis on four tetraploid plants obtained from the ‘OLL8’ somatic fusions with finger limes. Because flow cytometry cannot differentiate between allotetraploid and autotetraploid somatic fusions, EST-SSR marker analysis is needed to quickly verify the simple addition of two genomes resulting from the production of allotetraploid somatic hybrids, as expected during the somatic fusion process [38]. Six previously identified and well-characterized marker loci (CX0010, CX0035, CX2007, CX6F04, CX5F57, and CX6F29) were successfully utilized to generate a detailed allele (peak) table (Table 2). Our results revealed that all OLL8 + FL somatic hybrids obtained their alleles directly from their two donor parents by somatic addition and were therefore allotetraploids. EST-SSR markers have been specifically developed to allow the separation of homozygous and heterozygous loci [38]. The markers in the present study were selected because they are heterozygous in sweet orange [39] and there are no publicly available EST-SSR markers for the finger lime genome. Our results indicated that some markers developed for sweet oranges could also be used to confirm somatic hybrids containing the finger lime genome. Representative chromatograms of products obtained from PCR with the primer CX0010 are outlined in Fig 1D.

**Table 2:** Use of expressed sequence tag–simple sequence repeat (EST-SSR) to detect alleles and confirm allotetraploid somatic hybrids between OLL8 sweet orange and the finger lime (*C*. *australasica)*.

Parent/hybrids	EST-SSR primer amplified amplicon size (base pair)																	
	CX0010			CX0035				CX2007		CX6F04				CX5F57			CX6F29	
OLL8	220	230		144	146			170		163	174			165			152	154
Finger lime (FL)	220	223		147	148			197		144	157			150	160		154	154
OLL8+FL1	220	223	230	144	146	147	148	170	197	144	157	163	174	150	160	165	152	154
OLL8+FL2	220	223	230	144	146	147	148	170	197	144	157	163	174	150	160	165	152	154
OLL8+FL3	220	223	230	144	146	147	148	170	197	144	157	163	174	150	160	165	152	154
OLL8+FL4	220	223	230	144	146	147	148	170	197	144	157	163	174	150	160	165	152	154

### Organelle genome polymorphisms confirm the cybrid nature of diploid regenerants

**Fig 3:**
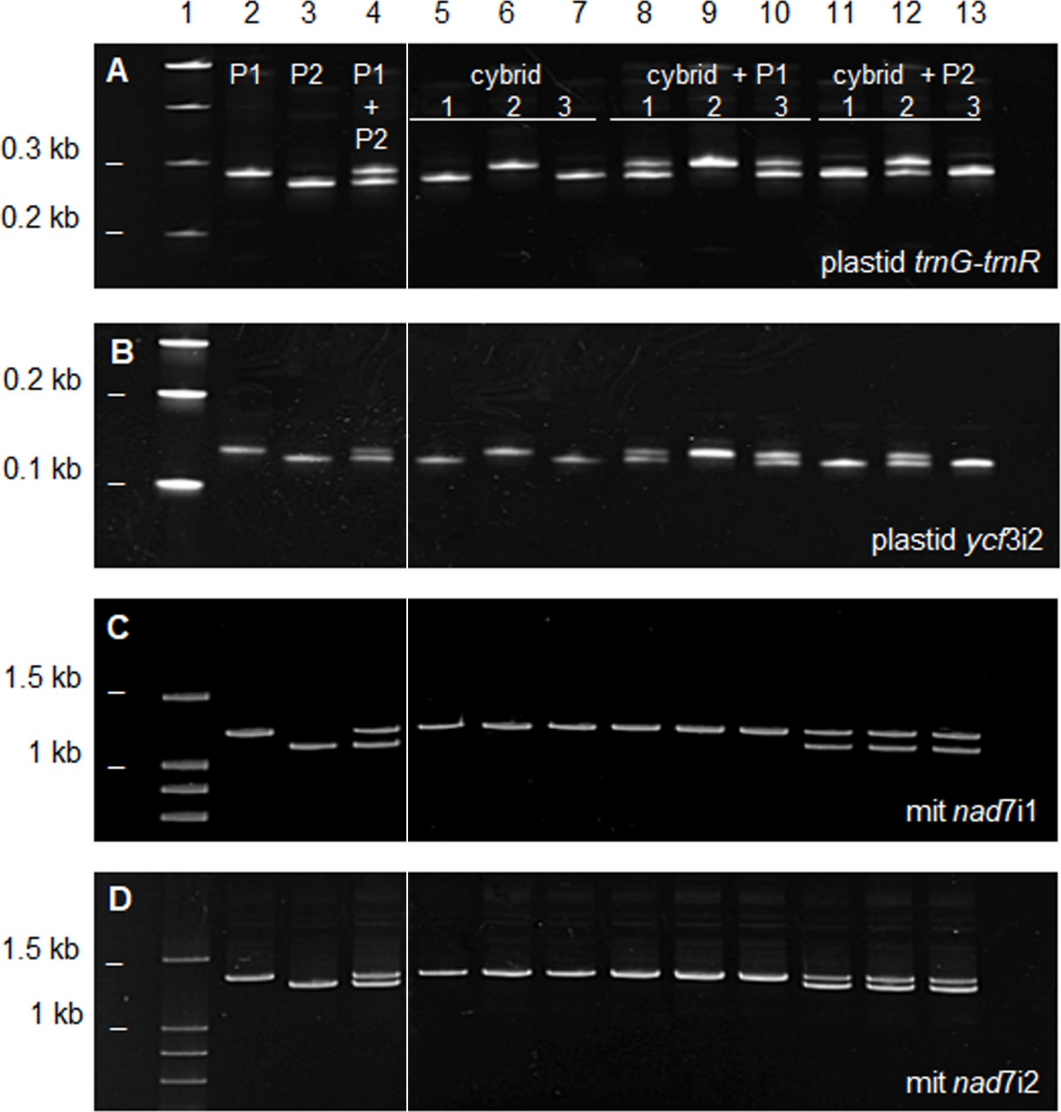
Organelle genome polymorphisms and inheritance in ‘Page’ mandarin and finger lime somatic cell fusion partners and products. PCR amplification products of fusion parent lines and fusion products are shown fractionated by polyacrylamide gel electrophoresis, stained with ethidium bromide and imaged on a Bio-Rad ChemiDoc™ Gel Imaging System. Panels A, B, C and D show the plastid *trnG*-*trnR* intergenic spacer, plastid ycf3i2, mitochondrial *nad*7i1 and mitochondrial *nad*7i2 amplification products, respectively. In all panels, lane 1 contains the Promega G210A 100 base pair marker ladder; lane 2 the ‘Page’ mandarin (P1) amplification product, lane 3 the finger lime (P2) amplification product and lane 4 a mixture of P1 and P2 amplifications, confirming length polymorphisms. Lanes 5–7 carry amplifications from three independent 2N fusion products. To confirm the cybrid nature of the 2N plants recovered following somatic cell fusion, amplification products of each were mixed with those of P1 (lanes 8–10) or P2 (11–13), demonstrating all to carry the P2 mitochondrial genome and either the P1 or P2 plastid genome. The white line after the lane 4 indicates the cropped area of the gel. Complete uncropped gel image is available as S1 Fig.

Plastid and mitochondrial DNA amplification products revealed length polymorphisms that distinguished the ‘Page’ mandarin and finger lime fusion partners. The plastid *trnG*-*trnR* intergenic region and *ycf3* intron 2 are both SSR markers in citrus [28] and the ‘Page’ mandarin amplification product is larger than that of the finger lime in both cases (Fig 3). Full-length amplification products of the mitochondrial *NADH dehydrogenase* subunit 7 intron 1 (*nad*7i1) and intron 2 (*nad*7i2) were also polymorphic for the fusion partners with ‘Page’ mandarin producing the longer product in both cases. DNA sequencing is required for accurate length determination because the length differences between amplicons are small. Electrophoresis of the mixed amplification products confirmed differences in amplicon size.

Organelle genome polymorphisms were expected based on studies of other citrus materials and the origins of the ‘Page’ mandarin and finger lime. ‘Page’ is a hybrid between a Minneola tangelo seed parent and a *C*. *reticulata* (mandarin) pollen parent. The Minneola tangelo itself is a hybrid between a *C*. *paradisi* (grapefruit) seed parent and a mandarin pollen parent [34]. Thus, the maternally inherited organelle genomes of ‘Page’ derive from grapefruit, which carries the *C*. *maxima* (pummelo) maternal lineage [40]. Recent analyses of multiple citrus genomes demonstrated that Australian limes, including *C*. *australasica* (finger lime), group more closely with mandarin and *C*. *japonica* (kumquat) and more distantly from pummelo. This information, combined with organelle inheritance studies in grapefruit-mandarin and grapefruit-kumquat somatic cell fusions [27,41], enabled the efficient selection of informative markers for the Page mandarin-finger lime combination.

### Organelle inheritance in somatic cell fusions

**Fig 4:**
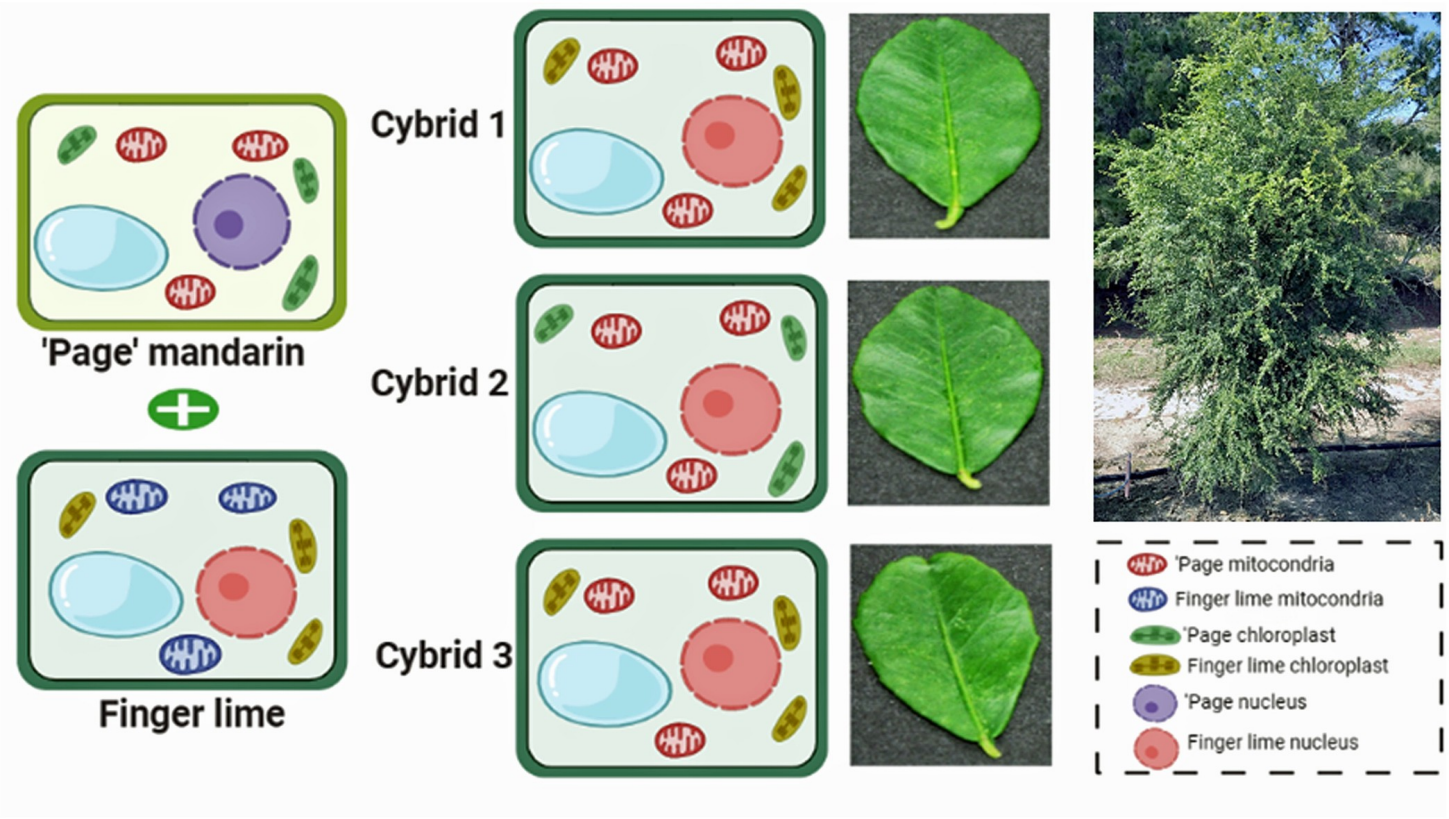
Schematic diagram showing nuclear and organelles inheritance of three cybrids through protoplast fusion. The cybrids plants are carrying the finger lime nuclear genotype. Two cybrids (1 and 3) inherited the finger lime plastids, while cybrid 2 inherited the ‘Page’ mandarin plastid genome. All finger lime cybrids inherited the ‘page’ mitochondrial genome. The top right tree represents one of the cybrids in the field. Figure was created in BioRender.com.

Polymorphic organelle DNA amplification products allow organelle genotyping of plants derived from somatic cell fusion events. All three independent diploid (2N) plants inherited only the ‘Page’ mandarin mitochondrial genome markers. Therefore, these 2N plants were cybrids carrying the finger lime nuclear genome combined with the ‘Page’ mandarin mitotype. The 2N fusion products varied with respect to the plastid genotype. Two 2N plants inherited the finger lime plastid type, whereas the remaining 2N plant inherited the ‘Page’ mandarin plastid genome (Figs 3 and 4).

The organelle genome inheritance patterns observed in *C*. *reticulata*-*C*. *australasica* somatic cell fusion products agree with previous observations for citrus, where mitochondrial genomes are generally derived from the cell suspension culture fusion partner, and the plastid genome is derived from either the suspension culture or leaf protoplast fusion partner [42]. This pattern was also observed in 4N somatic hybrids recovered from protoplasts of Carrizo citrange (*Citrus sinensis* × *Poncirus trifoliata*) mesophyll cells and *Citrus macrophylla* embryogenic callus [43]. These outcomes likely result from differences between the two cell types with respect to organelle morphology and genome copy number. Mitochondrial genome copy number is low (less than one per mitochondrion) in plant leaf cells [44]. In young leaf protoplasts, mitochondrial fusion likely consolidates genome information but drastically reduces the number of mitochondria [45]. In contrast, there is evidence of abundant plastids and plastid genomes in mesophyll protoplasts [46] and significant populations of replicating plastid and mitochondrial DNA molecules in suspension-cultured plant cells [47–50]. Because of the complex organization and active recombination of plant mitochondrial genomes [51,52], some contribution of the ‘Page’ mandarin mitochondrial genome sequences to the somatic hybrids and cybrids cannot be ruled out.

Predictable cybridization affords the opportunity to rapidly generate novel nuclear-organelle genome combinations that would take decades of conventional backcross breeding in perennial tree crops, such as citrus. This exchange of genomes can modify plant phenotypes in interesting and useful ways. Improved grapefruit quality over an extended harvest season is associated with the mandarin mitotype [41], whereas grapefruit trees carrying the kumquat plastid genotype have enhanced resistance to citrus canker disease [53]. Cybridization is a route to crops with novel juice and peel characteristics [54,55] and developing seedless fruit by incorporating mitochondrial genomes encoding pollen sterility traits [56,57]. Proteomics analysis of a ‘femminello’ lemon (*C*. *limon*)-‘Valencia’ sweet orange (*C*. *sinensis*) cybrid revealed the upregulation of proteins related to bioenergetics and stress tolerance, pointing to broad opportunities for improving plant performance through cybridization [58].

### Horticultural traits of the OLL8 + FL fusions

**Fig 5:**
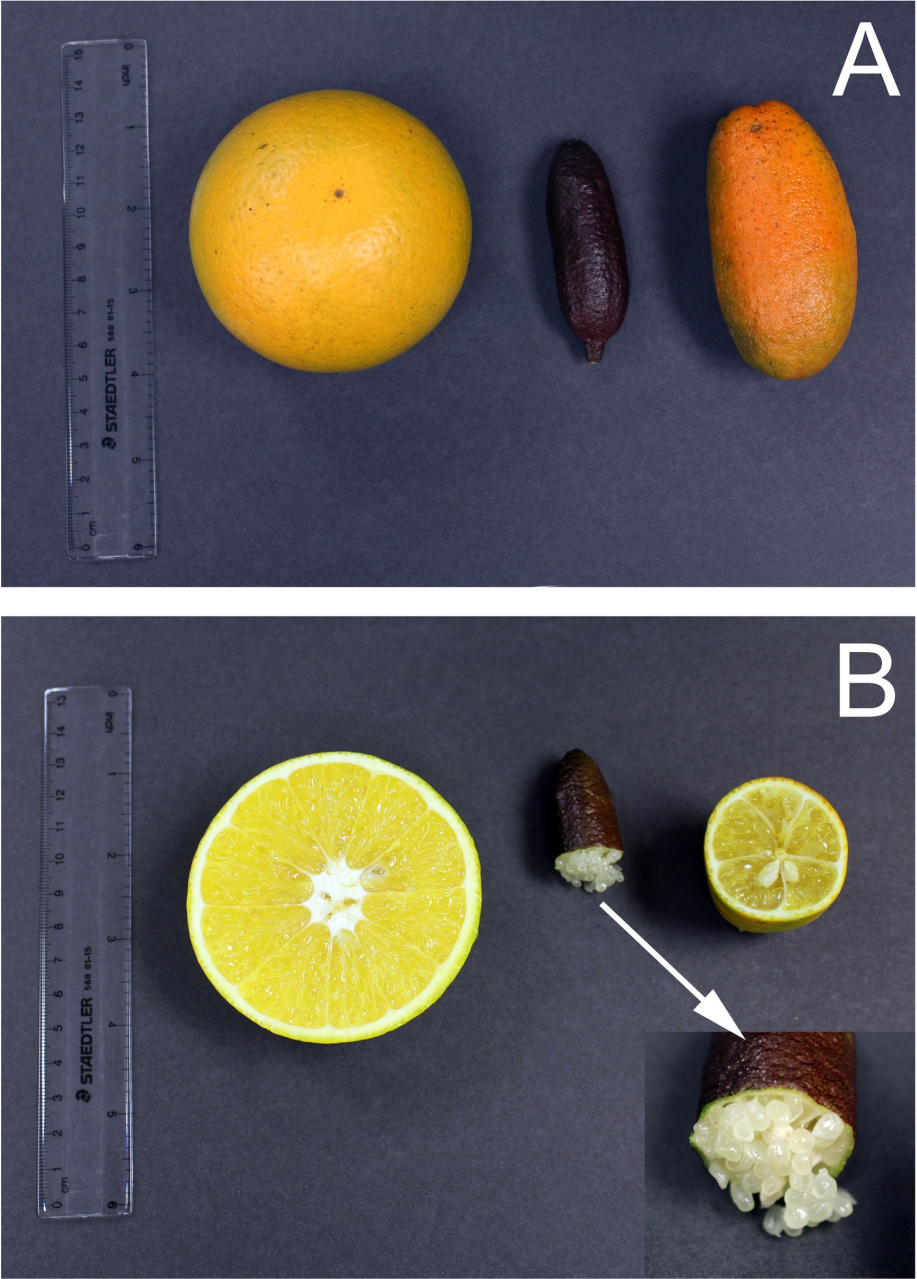
Fruit morphological characteristics of tetraploid finger lime and the diploid finger lime, external (A) and internal (B) characteristics. Left (OLL8, 2X), Middle (finger lime, 2X), right (OLL8+FL, 4X).

The OLL8 + FL tetraploid trees were small and compact, remaining between 3 and 4 feet in height after 6 years in the field. Trees were thorny, with leaves resembling the FL parent (Fig 1A). The leaves were dark green and strongly veined with small petioles. Flowers were borne singly on the leaf axils. Fruits matured during November-December and were yellow-orange in color when mature, resembling the OLL8 callus parent. Fruits were cylindric-fusiform in shape, resembling the FL mesophyll parent, but did not have a blunt protuberance at the blossom end, as seen in the FL parent (Fig 5). Fruits ranged from 7.5 to 8 cm in length with an average diameter of 3 to 3.5 cm. Fruit weighed from 48.7 to 57.2 g and contained an average of 4.8 to 6 seeds (Table 3). The pulp vesicles were pale yellow. Protoplast fusion among sour oranges and rough lemons with finger limes has been previously attempted [59]. However, no somatic fusion derived plants were generated in that study. Thus, our study provides novel information on the somatic fusion between a standard citrus and finger limes. The fruit shape in our somatic fusions resembled the ‘Minnie’ finger lime [60] but several other horticultural traits were unique.

**Table 3:** Average fruit characteristics of the different OLL8+FL somatic fusions.

Somatic fusion	Length (cm)	Diameter (cm)	Color	Fruit weight (grams)	Number of seeds	Number of embryo per seed
OLL8 + FL1	8.11 ± 1.1	3.04 ± 0.76	RHS 23A (yellow-orange)	57.2± 3.3	6.0 ± 1.1	50% mono embryonic
OLL8 + FL2	7.82 ± 0.75	3.50 ± 0.50	RHS 23A (yellow-orange)	52.1± 2.1	5.8 ± 0.4	100% mono embryonic
OLL8 + FL3	7.62 ± 1.52	3.55 ± 1.20	RHS 23A (yellow-orange)	57± 1.5	4.8 ± 0.9	100% mono embryonic
OLL8 + FL4	7.60 ± 1.77	3.10 ± 1.00	RHS 23A (yellow-orange)	48.7± 4.4	5.6 ± 0.5	100% mono embryonic

### Gene expression analysis revealed tetraploid plants to be similar to the HLB-tolerant finger lime parent

The tetraploid plants regenerated in this study were grafted onto Carrizo citrange rootstocks and planted in the field. The growth habits of the surviving lines were similar to those of the finger lime mesophyll parent, and the tetraploid plants had very little vegetative growth during the first 2 years after planting. The cybrid plants resembled the finger lime parent. Trees did not show any of the classic HLB symptoms of blotchy mottle conditions in the leaves, veinal chlorosis, or subsequent twig die-back [61,62]. Analysis of *Ca*Las DNA from the petiole and midrib from several 6-year-old allotetraploid OLL8 + finger lime somatic fusions and their diploid parents (of similar age) revealed that most somatic fusion lines and the finger lime parent were HLB negative even after prolonged exposure to an HLB-endemic environment (Table 4). Only the line 3 was observed to be HLB positive.

**Table 4:** Ct values of CaLas detected in the 6-year-old allotetraploid somatic hybrids between OLL8 sweet orange and the finger lime and the diploid parents.

Cultivar	Ct-value
OLL8	29.11 ± 0.80^b^
DPI-50-36	37.58 ± 0.44^a^
OLL+ FL 1	36.03 ± 0.25^a^
OLL+ FL 2	34.40 ± 0.41^a^
OLL+ FL 3	28.97 ± 0.14^b^
OLL+ FL 4	37.88 ± 0.08^a^

* Means separation by Tukey’s honestly significant difference test (P≤ 0.05).

**Fig 6:**
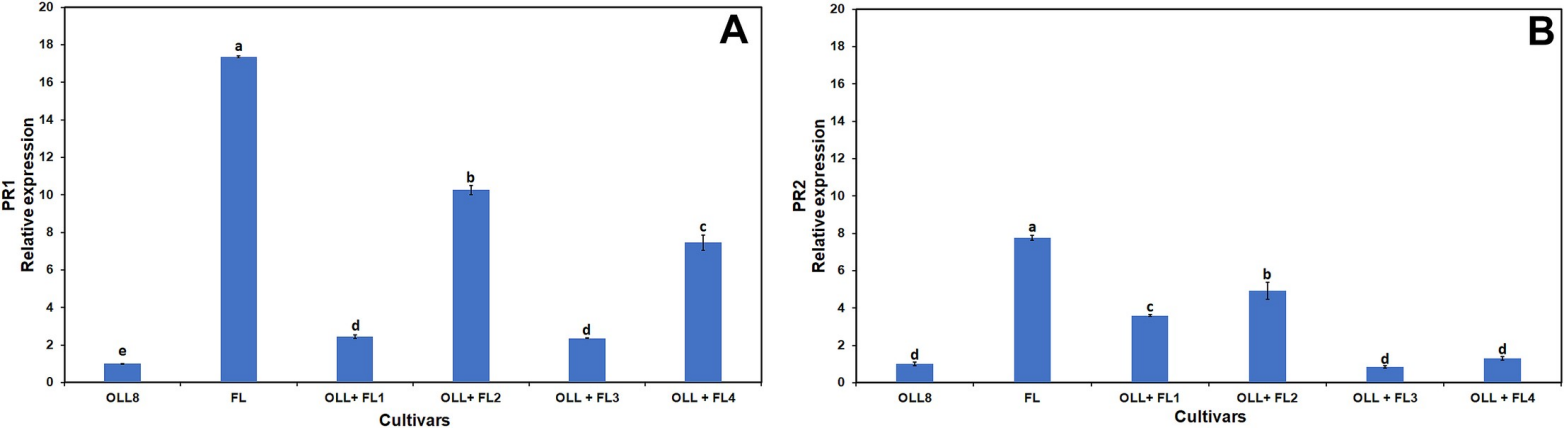
Relative expression of PR1 (A) and PR2 (B) transcripts in OLL8, finger lime (FL) and four selected OLL8+FL allotetraploids. Bars represent means ± standard error. Means separation by Tukey’s honestly significant difference test (P≤ 0.05).

Several genes are differentially expressed between susceptible and tolerant citrus following infection by *Ca*Las [30,63,64]. Additionally, several ion transport genes are differentially expressed between *Ca*Las*-*infected and healthy citrus trees [65]. The expression levels of PR1 and PR2 genes have long been used to gauge plant defense responses [66,67]. We observed enhanced expression of these genes in the finger lime mesophyll parent. Expression levels of the PR1 genes (Fig 6A) varied among the tested lines. Line 2 had the highest expression, followed by line 4. However, expression levels were lower than the FL mesophyll parent, but all lines had significantly enhanced expression when compared to the ‘OLL8’ callus parent. A similar trend was seen in PR2 expression with FL and all somatic fusion lines had significantly enhanced expression when compared to the ‘OLL8’ callus parent (Fig 6B). The PR1 protein is present in all plants [68] and is usually induced in response to pathogen attack [69]. Overexpression of the grapevine PR1 gene resulted in bacterial disease-tolerant transgenic tobacco, whereas the upregulated *Capsicum annuum* basic PR1 gene also demonstrated similar bacterial resistance in transgenic tobacco [70,71]. Thus, the PR1 protein plays a major role in disease resistance. In the present study, somatic fusion-derived OLL8 + FL fusion trees, as well as the parents, were grown under HLB-endemic conditions, and enhanced PR1 gene expression may have played a role in enhanced tolerance to HLB as observed earlier (Table 4). Additionally, PR2 transcripts were enhanced in the two somatic fusions and the FL parent. The PR2 gene encodes an acidic form of the β-1,3-glucanase protein and plays a role in the SAR process [72]. Transcripts are usually induced after fungal infection or wounding [73] but can also be induced along with the PR1 transcript [74].

**Fig 7:**
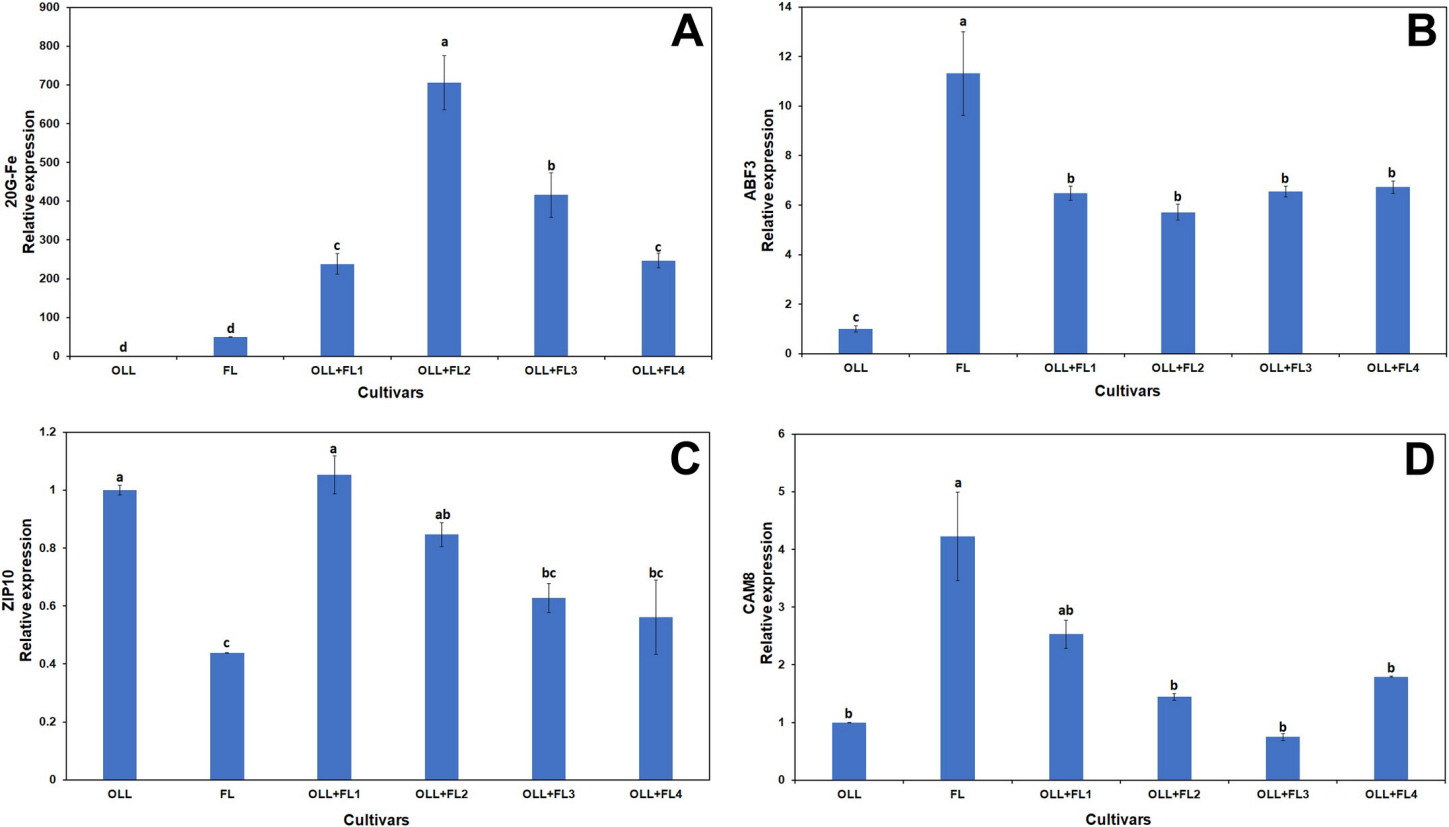
Relative expression of 20G-Fe (A), ABF3 (B), ZIP10 (C) and CAM8 (D) transcripts in OLL8, finger lime (FL) and four selected OLL8+FL allotetraploids. Bars represent means ± standard error. Means separation by Tukey’s honestly significant difference test (P≤ 0.05).

Additionally, we tested the expression of several other differentially regulated genes following infection with *Ca*Las (S3 Table). The 2-oxoglutarate/Fe(II)-dependent dioxygenase (2OG-Fe) transcript was highly upregulated in all somatic fusion lines, and the expression levels in the somatic fusion line 2 of the FL parent was more than 700-fold that of the ‘OLL8’ parental control (Fig 7A). Enhanced transcript accumulation was also observed in an auxin-responsive family protein (ABF3) with all somatic fusion lines, demonstrating statistically similar expression levels (Fig 7B). The zinc transporter 10 precursor (ZIP10) transcript levels were downregulated in the FL mesophyll parent, and all somatic fusion lines were statistically similar to the ‘OLL8’ callus parent (Fig 7C). The calcium-dependent calmodulin (CAM8) transcript levels were only statistically significant in the FL mesophyll parent (Fig 7D). The 2-oxoglutarate/Fe(II)-dependent dioxygenases (2OG-Fe) are a large group of oxidative enzymes that can catalyze many different plant metabolism reactions. These enzymes are known to function during DNA repair, histone methylation, post-translational modification, and iron sensing, as well as salicylic acid catabolism, among other activities [75]. Similarly, endogenous plant auxins play a major role in plant growth and development. Auxins are known to alter the expression of various genes [76] and play a role in plant defense [77]. Enhanced auxin levels can sometimes facilitate pathogenesis [78] but the selected gene (ABF3) evaluated in this study was downregulated in HLB-infected plants [65]. The CAM8 evaluated in this study was highly upregulated in the FL mesophyll parent and somatic hybrid line 1. In the other lines, expression was statistically similar to that of the OLL8 sweet orange control. Optimum intracellular calcium levels are crucial for activating plant-pathogen interactions that initiate local defense and SAR [79]. Ca^2+^-binding proteins, such as calmodulins, can sense and respond to fluctuations in intracellular Ca^2+^ levels [80]. Enhanced cellular calcium can produce firmer leaves that may not be attractive to sucking insects, such as the Asian citrus psyllid [81]. Neither the somatic fusions nor the parental lines demonstrated enhanced ZIP10 activity. This was in contrast with the results obtained by Shahzad et al. [65], who observed enhanced expression in HLB-infected citrus trees.

**Fig 8:**
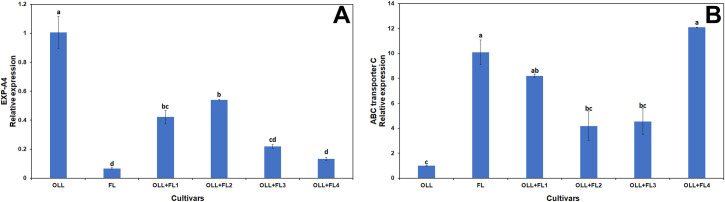
Relative expression of EXP-A4 (A) and ABC transporter C (B) transcripts in OLL8, finger lime (FL) and four selected OLL8+FL allotetraploids. Bars represent means ± standard error. Means separation by Tukey’s honestly significant difference test (P≤ 0.05).

We also tested the expression profile of a putative expansin gene (EXP-A4), which has been reported to be upregulated in HLB-infected citrus [64]. This gene was not significantly upregulated in any of the lines tested in this study (Fig 8A). Expansins regulate cell wall extension and are usually upregulated during cell growth [82]. The suppression of expansin genes can potentially promote resistance to pathogen invasion [83] by maintaining cell wall integrity. The ABC transporter family contains genes that shuttle substrates across biological membranes. These genes respond to abiotic or biotic stimuli [84] and allow plants to adapt to changing environments [85]. Enhanced transcript accumulation in the FL mesophyll parent and the four somatic fusions could help alleviate the HLB symptoms in plant cells through different mechanisms and initiate defense responses to counteract the damaging effects of *Ca*Las (Fig 8B).

## Conclusion

The development of novel citrus tetraploids has resulted in the establishment of a unique germplasm that could be a valuable resource for the genetic improvement of citrus. Most tetraploid trees remained HLB negative, indicating that the HLB tolerance trait from the FL mesophyll parent can be transmitted to the somatic fusions. This, in turn, can be potentially transmitted to the next generation of hybrids for the development of either HLB-tolerant triploid citrus scions or tetraploid citrus rootstocks. The gene expression profile indicating the upregulation of PR and other genes could have enhanced the plant defense response to HLB. Although we focused on OLL8 sweet orange fusions with finger lime, other fusions with mandarins and lemons can also be potentially produced. Additionally, the cybrids produced in this study resulted in the production of novel intergenomic recombinations that are not possible through conventional breeding techniques.

## Supporting information

S1 Fig Original uncropped images of composite Fig 3. Dotted lines indicate area cropped out from each gel.
(PDF)

S1 Table List of the primer sequences used for SSR marker analysis. (PDF)

S2 Table List of primers generating polymorphic organelle genome amplification products. (PDF)

S3 Table TAQMAN based primer sequences used to amplify a 87-bp fragment of the *Ca*Las rplJ/rplL ribosomal protein gene. (PDF)

S4 Table List of the primer sequences used in SYBR Green based real-time PCR assay. (PDF)

## References

[1] Mahmoud LM, Grosser JW, Dutt M. Silver compounds regulate leaf drop and improve in vitro regeneration from mature tissues of Australian finger lime (Citrus australasica). Plant Cell Tissue Organ Cult. 2020; 141: 455–464.

[2] Fahn A, Shomer I, Ben-Gera I. Occurrence and structure of epicuticular wax on the juice vesicles of citrus fruits. Ann Bot. 1974; 38:869–872.

[3] Ramadugu C, Keremane ML, Halbert SE, Duan YP, Roose ML, Stover E, et al. Long-Term Field Evaluation Reveals Huanglongbing Resistance in Citrus Relatives. Plant Dis. 2016;100:1858–1869. doi: 10.1094/PDIS-03-16-0271-RE 30682983

[4] Folimonova SY, Robertson CJ, Garnsey SM, Gowda S, Dawson WO. Examination of the responses of different genotypes of citrus to huanglongbing (citrus greening) under different conditions. Phytopathology. 2009;99:1346–1354. doi: 10.1094/PHYTO-99-12-1346 19900000

[5] de Wit PJ. How plants recognize pathogens and defend themselves. Cell Mol Life Sci. 2007; 64: 2726–2732. doi: 10.1007/s00018-007-7284-7 17876517 PMC11136299

[6] Stover E, Gmitter FG, Grosser J, Baldwin E, Wu GA, Bai J, et al. Rationale for reconsidering current regulations restricting use of hybrids in orange juice. Hortic Res. 2020; 7: 1–7. doi: 10.1038/s41438-019-0222-7 32194974 PMC7060986

[7] Stover E, McCollum G. Incidence and severity of huanglongbing and Candidatus Liberibacter asiaticus titer among field-infected citrus cultivars. HortScience. 2011;46:1344–1348.

[8] McCollum G, Hilf M, Irey M, Luo W, Gottwald T. Susceptibility of sixteen citrus genotypes to ‘Candidatus Liberibacter asiaticus’. Plant Dis. 2016;100:1080–1086. doi: 10.1094/PDIS-08-15-0940-RE 30682269

[9] Stover E, McCollum GT, Driggers R, Lee R, Shatters R Jr, Duan Y, et al. Resistance and tolerance to Huanglongbing in citrus. Acta Hortic. 2015;1065:899–903.

[10] Deng H, Achor D, Exteberria E, Yu Q, Du D, Stanton D, et al. Phloem regeneration is a mechanism for Huanglongbing-tolerance of “Bearss” lemon and “LB8-9” Sugar Belle® mandarin. Front Plant Sci. 2019;10:277. doi: 10.3389/fpls.2019.00277 30949186 PMC6435995

[11] Forner-Giner MA, Continella A, Grosser JW. Citrus Rootstock Breeding and Selection. The Citrus Genome: Springer. 2020; 49–74.

[12] Germanà MA, Aleza P, Grosser JW, Dutt M, Wang N, Cuenca J, et al. Citrus biotechnology. The Genus Citrus: Elsevier. 2020; 171–192.

[13] Ćalović M, Chen C, Yu Q, Orbović V, Gmitter FG, Grosser JW. New Somatic Hybrid Mandarin Tetraploids Generated by Optimized Protoplast Fusion and Confirmed by Molecular Marker Analysis and Flow Cytometry. J Am Soc Hortic Sci. 2019;144:151–163.

[14] Sattler MC, Carvalho CR, Clarindo WR. The polyploidy and its key role in plant breeding. Planta. 2016;243:281–296. doi: 10.1007/s00425-015-2450-x 26715561

[15] Ollitrault P, Germanà MA, Froelicher Y, Cuenca J, Aleza P, Morillon R, et al. Ploidy Manipulation for Citrus Breeding, Genetics, and Genomics. The Citrus Genome: Springer. 2020;75–105.

[16] Adams KL, Wendel JF. Polyploidy and genome evolution in plants. Curr Opin Plant Biol. 2005;8:135–141. doi: 10.1016/j.pbi.2005.01.001 15752992

[17] Grosser J, Mourao-Filho F, Gmitter F, Louzada E, Jiang J, Baergen K, et al. Allotetraploid hybrids between Citrus and seven related genera produced by somatic hybridization. Theor Appl Genet. 1996;92:577–582. doi: 10.1007/BF00224561 24166326

[18] Dutt M, Vasconcellos M, Song K, Gmitter F, Grosser J. In vitro production of autotetraploid Ponkan mandarin (Citrus reticulata Blanco) using cell suspension cultures. Euphytica. 2010;173:235–242.

[19] Grosser JW. Citrus rootstock named ‘UFR-4’. US Patent USPP27745P3. 2017.

[20] Stover E, Hall DG, Grosser J, Gruber B, Moore GA. Huanglongbing-related Responses of ‘Valencia’ Sweet Orange on Eight Citrus Rootstocks during Greenhouse Trials. HortTechnology. 2018;28:776–782.

[21] Grosser JW, Gmitter FG. Protoplast fusion for production of tetraploids and triploids: applications for scion and rootstock breeding in citrus. Plant Cell Tissue Organ Cult. 2011;104:343–357.

[22] Grosser JW, An HJ, Calovic M, Lee DH, Chen C, Vasconcellos M, et al, Production of new allotetraploid and autotetraploid citrus breeding parents: focus on zipperskin mandarins. HortScience. 2010;45:1160–1163.

[23] Grosser JW, Calovic M, Louzada ES. Protoplast fusion technology-somatic hybridization and cybridization. Plant Cell Culture, John Wiley & Sons, Ltd. 2010:175–198.

[24] Grosser JW, Ollitrault P, Olivares-Fuster O. Somatic hybridization in citrus: an effective tool to facilitate variety improvement. In Vitro Cell Dev Biol-Plant 2000; 36: 434–449.

[25] Grosser JW, Gmitter FG, Chandler J, Louzada ES. Somatic hybridization of complementary citrus rootstock: five new hybrids. HortScience. 1994;29:812–813.

[26] Grosser JW, Gmitter Jr FG. Protoplast fusion and citrus improvement. Plant Breed Rev. 1990;8:339–374.

[27] Omar AA, Murata M, Yu Q, Gmitter FG, Chase CD, Graham JH, et al. Production of three new grapefruit cybrids with potential for improved citrus canker resistance. In Vitro Cell Dev Biol-Plant. 2017; 53: 256–269.

[28] Cheng Y, De Vicente MC, Meng H, Guo W, Tao N, Deng X. A set of primers for analyzing chloroplast DNA diversity in Citrus and related genera. Tree Physiol. 2005;25:661–672. doi: 10.1093/treephys/25.6.661 15805086

[29] Wang Z, Yin Y, Hu H, Yuan Q, Peng G, Xia Y. Development and application of molecular‐based diagnosis for ‘Candidatus Liberibacter asiaticus’, the causal pathogen of citrus huanglongbing. Plant Pathol. 2006; 55: 630–638.

[30] Qiu W, Soares J, Pang Z, Huang Y, Sun Z, Wang N, et al. Potential Mechanisms of AtNPR1 Mediated Resistance against Huanglongbing (HLB) in Citrus. Int J Mol Sci. 2020;21: 2009. 10.3390/ijms21062009 PMC7139736 32187998

[31] Livak KJ, Schmittgen TD. Analysis of relative gene expression data using real-time quantitative PCR and the 2(-Delta Delta C(T)) Method. Methods. 2001; 25: 402–408. doi: 10.1006/meth.2001.1262 11846609

[32] Xu Q, Chen LL, Ruan X, Chen D, Zhu A, Chen C, et al. The draft genome of sweet orange (Citrus sinensis). Nat Genet. 2013; 45: 59–66. doi: 10.1038/ng.2472 23179022

[33] Grosser JW. Sweet orange tree named ‘OLL-8’. US Patent USPP26087P3. 2015.

[34] Reece PC, Hearn CJ, Gardner FE. Page orange- a promising variety. Proc Fla State Hortic Soc. 1963; 76:53–54.

[35] Grosser J, Gmitter F, Tusa N, Chandler J. Somatic hybrid plants from sexually incompatible woody species: Citrus reticulata and Citropsis gilletiana. Plant Cell Rep. 1990; 8: 656–659. doi: 10.1007/BF00269986 24232779

[36] Liqin G, Jianguo Z, Xiaoxia L, Guodong R. Polyploidy-related differential gene expression between diploid and synthesized allotriploid and allotetraploid hybrids of Populus. Molecular Breed. 2019; 39: 69.

[37] Tan FQ, Tu H, Liang WJ, Long JM, Wu XM, Zhang HY, et al. Comparative metabolic and transcriptional analysis of a doubled diploid and its diploid citrus rootstock (C. junos cv. Ziyang xiangcheng) suggests its potential value for stress resistance improvement. BMC Plant Biol. 2015;15: 89. doi: 10.1186/s12870-015-0450-4 25848687 PMC4374211

[38] Chen C, Grosser JW, Ćalović M, Serrano P, Pasquali G, Gmitter J, et al. Verification of Mandarin and Pummelo somatic hybrids by expressed sequence tag–simple sequence repeat marker analysis. J Am Soc Hortic Sci. 2008;133: 794–800.

[39] Chen C, Bowman KD, Choi YA, Dang PM, Rao MN, Huang S, et al. EST-SSR genetic maps for Citrus sinensis and Poncirus trifoliata. Tree Genet Genomes. 2008; 4:1–10.

[40] Wu GA, Terol J, Ibanez V, López-García A, Pérez-Román E, Borredá C, et al. Genomics of the origin and evolution of Citrus. Nature. 2018;554:311–316. doi: 10.1038/nature25447 29414943

[41] Satpute AD, Chen C, Gmitter FG, Ling P, Yu Q, Grosser MR, et al. Cybridization of grapefruit with ‘Dancy’ mandarin leads to improved fruit characteristics. J Am Soc Hortic Sci. 2015;140: 427–435.

[42] Guo WW, Xiao SX, Deng XX. Somatic cybrid production via protoplast fusion for citrus improvement. Scientia Hortic. 2013;163:20–26.

[43] Ruiz M, Pensabene-Bellavia G, Quiñones A, García-Lor A, Morillon R, Ollitrault P, et al. 2018. Molecular characterization and stress tolerance evaluation of new allotetraploid somatic hybrids between carrizo citrange and Citrus macrophylla W. rootstocks. Front Plant Sci. 2018; 9: 901. doi: 10.3389/fpls.2018.00901 30123223 PMC6085489

[44] Preuten T, Cincu E, Fuchs J, Zoschke R, Liere K, Börner T. Fewer genes than organelles: extremely low and variable gene copy numbers in mitochondria of somatic plant cells. Plant J. 2010; 64: 948–959. doi: 10.1111/j.1365-313X.2010.04389.x 21143676

[45] Sheahan MB, McCurdy DW, Rose RJ. Mitochondria as a connected population: ensuring continuity of the mitochondrial genome during plant cell dedifferentiation through massive mitochondrial fusion. Plant J. 2005; 44: 744–755. doi: 10.1111/j.1365-313X.2005.02561.x 16297067

[46] Sakamoto W, Takami T. Chloroplast DNA dynamics: copy number, quality control and degradation. Plant Cell Physiol. 2018; 59:1120–1127. doi: 10.1093/pcp/pcy084 29860378

[47] Backert S, Lurz R, Börner T. Electron microscopic investigation of mitochondrial DNA fromChenopodium album (L.). Curr Genet. 1996; 29: 427–436. doi: 10.1007/BF02221510 8625421

[48] Heinhorst S, Cannon G, Weissbach A. Plastid and nuclear DNA synthesis are not coupled in suspension cells of Nicotiana tabacum. Plant Mol Biol. 1985; 4: 3–12. doi: 10.1007/BF02498710 24310651

[49] Takeda Y, Hirokawa H, Nagata T. The replication origin of proplastid DNA in cultured cells of tobacco. Mol Genet Genom. 1992; 232: 191–198. 10.1007/BF00279996 1557025

[50] Wang Y, Saitoh Y, Sato T, Hidaka S, Tsutsumi KI. Comparison of plastid DNA replication in different cells and tissues of the rice plant. Plant Mol Biol. 2003; 52: 905–913. doi: 10.1023/a:1025093528174 13677476

[51] Gualberto JM, Newton KJ. Plant mitochondrial genomes: dynamics and mechanisms of mutation. Annu Rev Plant Biol. 2017; 68: 225–252. doi: 10.1146/annurev-arplant-043015-112232 28226235

[52] Morley SA, Nielsen BL. Plant mitochondrial DNA. Molecules. 2017; 15: 17. doi: 10.2741/4531 27814661

[53] Murata MM, Omar AA, Mou Z, Chase CD, Grosser JW, Graham JH. Novel plastid-nuclear genome combinations enhance resistance to citrus canker in cybrid grapefruit. Front Plant Sci. 2019; 9: 1858. doi: 10.3389/fpls.2018.01858 30666259 PMC6330342

[54] Abbate L, Tusa N, Del Bosco SF, Strano T, Renda A, Ruberto G. Genetic improvement of Citrus fruits: New somatic hybrids from Citrus sinensis (L.) Osb. and Citrus limon (L.) Burm. F. Food Res Int. 2012; 48: 284–290.

[55] Del Bosco SF, Napoli E, Mercati F, Abbate L, Carimi F, Ruberto G. Somatic cybridization for Citrus: polyphenols distribution in juices and peel essential oil composition of a diploid cybrid from Cleopatra mandarin (Citrus reshni Hort. ex Tan.) and sour orange (Citrus aurantium L.). Genet Resour Crop Evol. 2017; 64: 261–275.

[56] Xiao SX, Biswas MK, Li MY, Deng XX, Xu Q, Guo WW. Production and molecular characterization of diploid and tetraploid somatic cybrid plants between male sterile Satsuma mandarin and seedy sweet orange cultivars. Plant Cell Tissue Organ Cult. 2014; 116: 81–88.

[57] Zheng BB, Wu XM, Ge XX, Deng XX, Grosser JW, Guo WW. Comparative transcript profiling of a male sterile cybrid pummelo and its fertile type revealed altered gene expression related to flower development. PLoS One. 2012; 7: e43758. doi: 10.1371/journal.pone.0043758 22952758 PMC3429507

[58] Faddetta T, Abbate L, Renzone G, Piccionello AP, Maggio A, Oddo E, et al. An integrated proteomic and metabolomic study to evaluate the effect of nucleus-cytoplasm interaction in a diploid citrus cybrid between sweet orange and lemon. Plant Mol Biol. 2018; 98: 407–425. doi: 10.1007/s11103-018-0787-9 30341661

[59] Vardi A, Arzee-Gonen P, Frydman-Shani A, Bleichman S, Galun E. Protoplast-fusion-mediated transfer of organelles from Microcitrus into Citrus and regeneration of novel alloplasmic trees. Theor Appl Genet.. 1989; 78: 741–747. doi: 10.1007/BF00262572 24225837

[60] Bowman KD, McCollum G, Plotto A, Bai J. Minnie Finger Lime: A New Novelty Citrus Cultivar. HortScience. 2019;54: 1425–1428.

[61] Bové JM. Huanglongbing: a destructive, newly-emerging, century-old disease of citrus. J Plant Pathol. 2006: 7–37.

[62] da Graça J, Kunta M, Sétamou M, Rascoe J, Li W, Nakhla MK, et al. Huanglongbing in Texas: Report on the first detections in commercial citrus. J Citrus Pathol. 2015; 2(1).

[63] Albrecht U, Bowman KD. Transcriptional response of susceptible and tolerant citrus to infection with Candidatus Liberibacter asiaticus. Plant Sci. 2012; 185:118–130. doi: 10.1016/j.plantsci.2011.09.008 22325873

[64] Albrecht U, Bowman KD. Gene expression in Citrus sinensis (L.) Osbeck following infection with the bacterial pathogen Candidatus Liberibacter asiaticus causing Huanglongbing in Florida. Plant Sci. 2008; 175: 291–306.

[65] Shahzad F, Chun C, Schumann A, Vashisth T. Nutrient Uptake in Huanglongbing-affected Sweet Orange: Transcriptomic and Physiological Analysis. J Am Soc Hortic Sci. 2020; 145: 349–362.

[66] Mitsuhara I, Iwai T, Seo S, Yanagawa Y, Kawahigasi H, Hirose S, et al. Characteristic expression of twelve rice PR1 family genes in response to pathogen infection, wounding, and defense-related signal compounds (121/180). Mol Genet Genom. 2008;279: 415–427. 10.1007/s00438-008-0322-9 PMC2270915 18247056

[67] Le Henanff G, Heitz T, Mestre P, Mutterer J, Walter B, Chong J. Characterization of Vitis vinifera NPR1 homologs involved in the regulation of pathogenesis-related gene expression. BMC Plant Biol. 2009; 9: 54. doi: 10.1186/1471-2229-9-54 19432948 PMC2686700

[68] Lincoln JE, Sanchez JP, Zumstein K, Gilchrist DG. Plant and animal PR1 family members inhibit programmed cell death and suppress bacterial pathogens in plant tissues. Mol Plant Pathol. 2018;19:2111–2123. doi: 10.1111/mpp.12685 29603552 PMC6638019

[69] van Loon LC, Rep M, Pieterse CM. Significance of inducible defense-related proteins in infected plants. Annu Rev Phytopathol. 2006;44:135–162. doi: 10.1146/annurev.phyto.44.070505.143425 16602946

[70] Sarowar S, Kim YJ, Kim EN, Kim KD, Hwang BK, Islam R, et al. Overexpression of a pepper basic pathogenesis-related protein 1 gene in tobacco plants enhances resistance to heavy metal and pathogen stresses. Plant Cell Rep. 2005;24: 216–224. doi: 10.1007/s00299-005-0928-x 15719238

[71] Li ZT, Dhekney SA, Gray DJ. PR-1 gene family of grapevine: a uniquely duplicated PR-1 gene from a Vitis interspecific hybrid confers high level resistance to bacterial disease in transgenic tobacco. Plant Cell Rep. 2011;30: 1–11. doi: 10.1007/s00299-010-0934-5 20967449

[72] Ali S, Ganai BA, Kamili AN, Bhat AA, Mir ZA, Bhat JA, et al. Pathogenesis-related proteins and peptides as promising tools for engineering plants with multiple stress tolerance. Microbiol Res. 2018; 212: 29–37. doi: 10.1016/j.micres.2018.04.008 29853166

[73] Ali S, Chandrashekar N, Rawat S, Nayanakantha N, Mir Z, Manoharan A, et al. Isolation and molecular characterization of pathogenesis related PR2 gene and its promoter from Brassica juncea. Biol Plant 2017; 61: 763–773.

[74] Chen YL, Lee CY, Cheng KT, Chang WH, Huang RN, Nam HG, et al. Quantitative peptidomics study reveals that a wound-induced peptide from PR-1 regulates immune signaling in tomato. Plant Cell. 2014; 26: 4135–4148. doi: 10.1105/tpc.114.131185 25361956 PMC4247587

[75] Farrow SC, Facchini PJ. Functional diversity of 2-oxoglutarate/Fe(II)-dependent dioxygenases in plant metabolism. Front Plant Sci. 2014;5:524. doi: 10.3389/fpls.2014.00524 25346740 PMC4191161

[76] Jain M, Kaur N, Tyagi AK, Khurana JP. The auxin-responsive GH3 gene family in rice (Oryza sativa). Funct Integr Genomics. 2005; 6: 36. doi: 10.1007/s10142-005-0142-5 15856348

[77] Vidhyasekaran P. Plant hormone signaling systems in plant innate immunity. 2015.

[78] Kunkel BN, Harper CP. The roles of auxin during interactions between bacterial plant pathogens and their hosts. J Exp Bot. 2018; 69: 245–254. doi: 10.1093/jxb/erx447 29272462

[79] Lecourieux D, Ranjeva R, Pugin A. Calcium in plant defence‐signalling pathways. New Phytol. 2006;171: 249–269. doi: 10.1111/j.1469-8137.2006.01777.x 16866934

[80] Bergey D, Kandel R, Tyree B, Dutt M, Dhekney S. The role of calmodulin and related proteins in plant cell function: an ever-thickening plot. Springer Sci Rev. 2014;2:145–159.

[81] Eticha D, Kwast A, de Souza Chiachia TR, Horowitz N, Stützel H. Calcium nutrition of orange and its impact on growth, nutrient uptake and leaf cell wall Citrus Res Technol. 2017; 38: 62–70.

[82] Li Y, Jones L, McQueen-Mason S. Expansins and cell growth. Curr Opin Plant Biol. 2003; 6: 603–610. doi: 10.1016/j.pbi.2003.09.003 14611960

[83] Wang Y, Zhou L, Yu X, Stover E, Luo F, Duan Y. Transcriptome profiling of Huanglongbing (HLB) tolerant and susceptible citrus plants reveals the role of basal resistance in HLB tolerance. Front Plant Sci. 2016; 7: 933. doi: 10.3389/fpls.2016.00933 27446161 PMC4923198

[84] Lin CY, Trinh NN, Lin CW, Huang HJ. Transcriptome analysis of phytohormone, transporters and signaling pathways in response to vanadium stress in rice roots. Plant Physiol Biochem 2013; 66: 98–104. doi: 10.1016/j.plaphy.2013.02.007 23500712

[85] Sharom FJ, Kretzschmar T, Burla B, Lee Y, Martinoia E, Nagy R. Functions of ABC transporters in plants. Essays Biochem. 2011; 50: 145–160. doi: 10.1042/bse0500145 21967056

