# Supplementary material for: Utilization of somatic fusion techniques for the development of HLB tolerant breeding resources employing the Australian finger lime (Citrus australasica)
Source: PLoS One. 2021 Aug 10;16(8):e0255842. doi: 10.1371/journal.pone.0255842 (PMC8354479; doi:10.1371/journal.pone.0255842)

S1 Fig. Original uncropped images of composite Fig 3. Dotted lines indicate area cropped out from each gel.

Top gel panel: Gel following PCR amplification with primers for plastid *trnG-trnR*

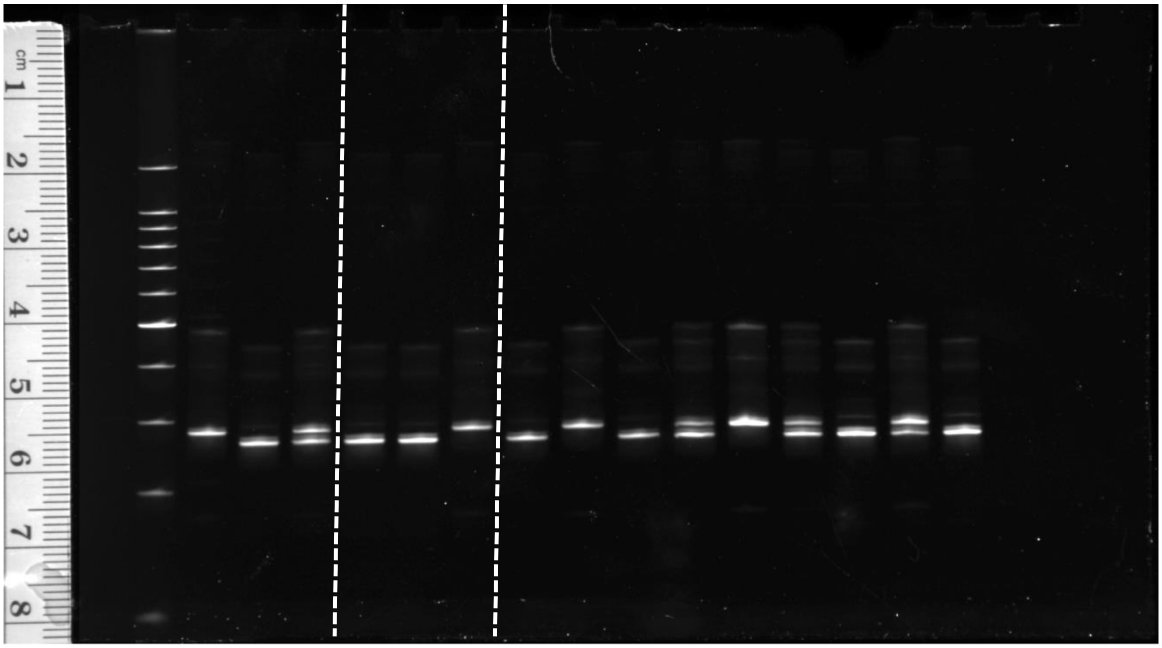

Second gel panel: Gel following PCR amplification with primers for plastid *ycf3i2*

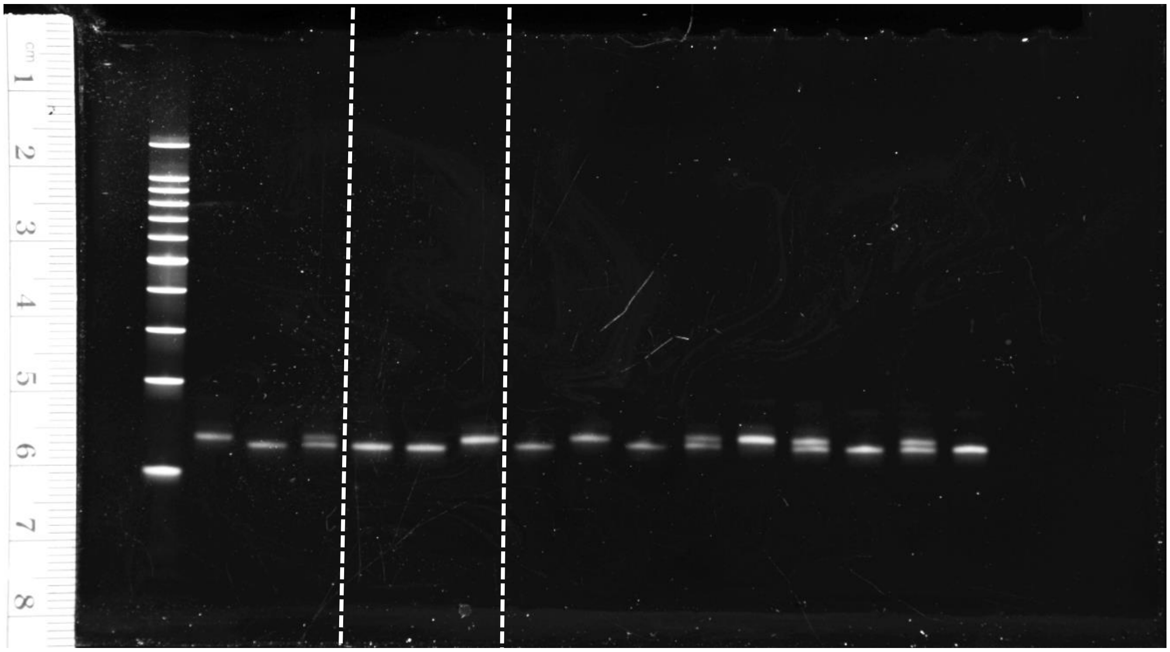

Third gel panel: Gel following PCR amplification with primers for mit *nad7i1*

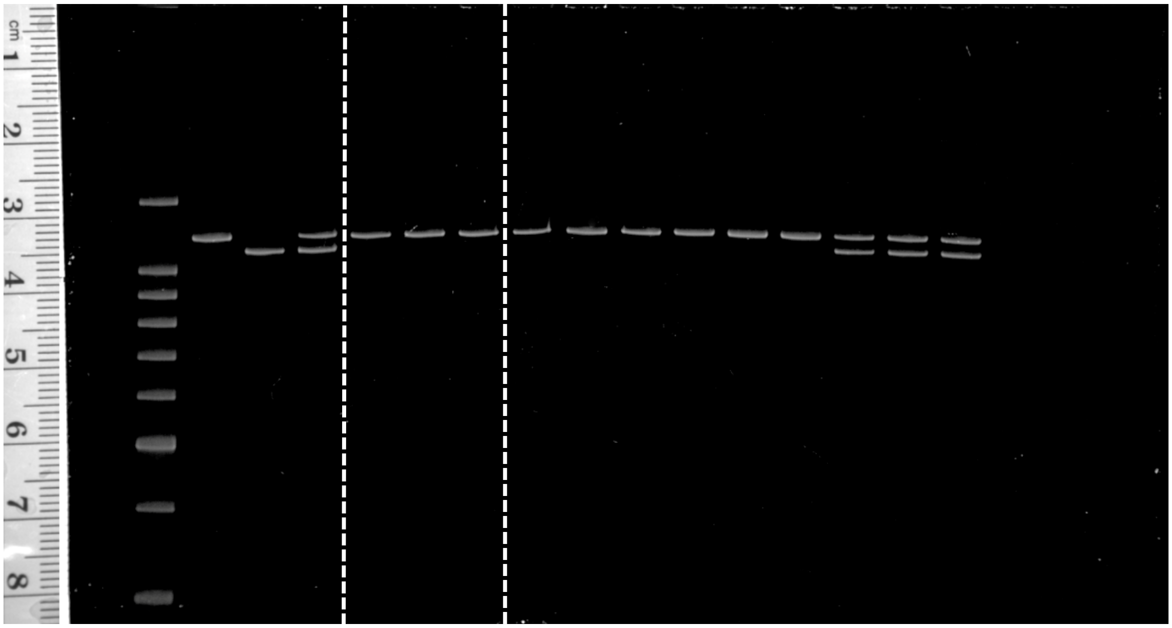

Bottom gel panel: Gel following PCR amplification with primers for mit *nad7i2*

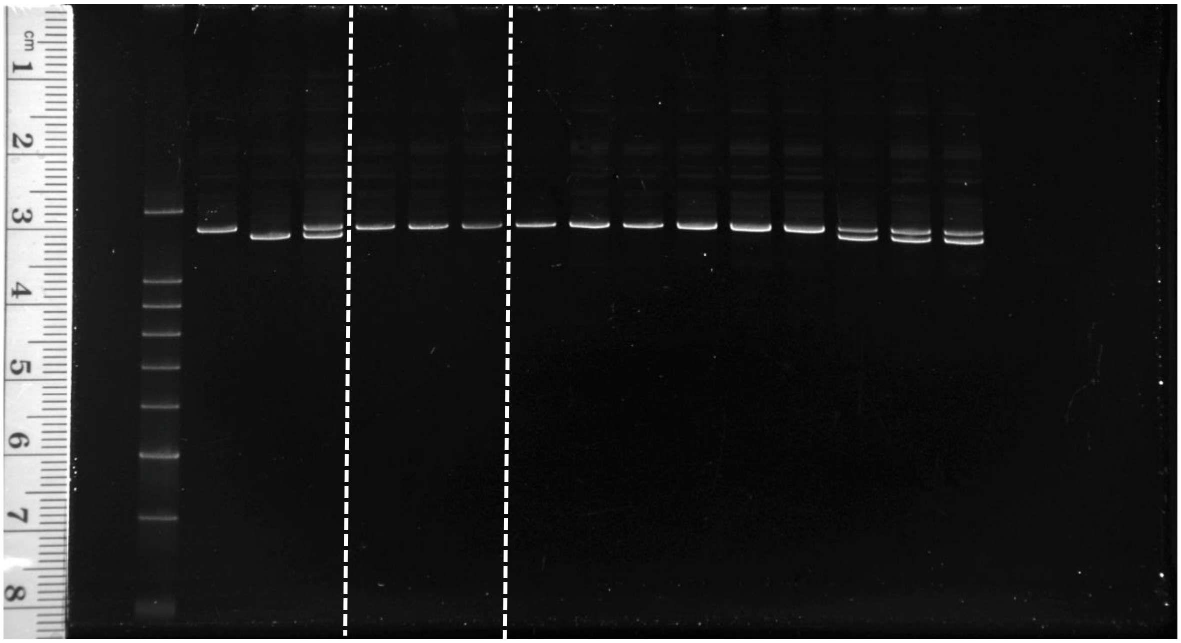

Supplement: S1 Fig — Dotted lines indicate area cropped out from each gel. (PDF) [file pone.0255842.s001.pdf]
