# Supplementary material for: Utilization of somatic fusion techniques for the development of HLB tolerant breeding resources employing the Australian finger lime (Citrus australasica)
Source: PLoS One. 2021 Aug 10;16(8):e0255842. doi: 10.1371/journal.pone.0255842 (PMC8354479; doi:10.1371/journal.pone.0255842)
Supplement: S1 Table — (PDF) [file pone.0255842.s002.pdf]

S1 Table. List of the primer sequences used for SSR marker analysis.

Primer	Forward and reverse primer sequences (5’ to 3’)
CX6F04	AGTGAACTGTCCATTGGATTTTCG<br>GTGTTGAATCCCGACCTTCTACC
CX6F29	TTCACCACAAACGAAGACTCAGAC<br>CTGTAATCCACTCGGTAATCCGAC
CX5F57	CCTCGCCAATGACCTTTGTATTTA<br>CAATACGTTTGGGTTCTAGTTCCG
CX0010	AACCGAAGATGGAGGGAACT<br>ACATTCATGGCCACATCTCA
CX0035	CCATTAACGAGAAAACCAAACA<br>CAAAAAGGGGTTGCAAAGAA
CX2007	AAATCGGCTAGTTGCAAACG<br>CCTTGACATTGTCGATGGTG
