# Supplementary material for: Utilization of somatic fusion techniques for the development of HLB tolerant breeding resources employing the Australian finger lime (Citrus australasica)
Source: PLoS One. 2021 Aug 10;16(8):e0255842. doi: 10.1371/journal.pone.0255842 (PMC8354479; doi:10.1371/journal.pone.0255842)
Supplement: S2 Table — (PDF) [file pone.0255842.s003.pdf]

S2 Table. List of primers generating polymorphic organelle genome amplification products.

Marker	Target	Forward and reverse primer sequences (5' to 3')
CCMP6	plastid<br>*ycf3* intron 2	CGATGCATATGTAGAAAGCC<br>CATTACGTGCGACTATCTCC
NTCP9	plastid<br>*trnG-trnR* IR[b]	CTTCCAAGCTAACGATGC<br>CTGTCCTATCCATTAGACAATG
*nad*7i1	mitochondria<br>*nad*7 intron 1	AACGGAGAAGTGGTGGAACG<br>TTTCTCAGTCCCTCTAGTCG
*nad*7i2	mitochondria<br>*nad*7 intron 2	AGATGCCAGCGGAATGAT<br>GTGTTCTTGGGCCATCATAG
