# Supplementary material for: Utilization of somatic fusion techniques for the development of HLB tolerant breeding resources employing the Australian finger lime (Citrus australasica)
Source: PLoS One. 2021 Aug 10;16(8):e0255842. doi: 10.1371/journal.pone.0255842 (PMC8354479; doi:10.1371/journal.pone.0255842)
Supplement: S3 Table — (PDF) [file pone.0255842.s004.pdf]

S3 Table. TAQMAN based primer sequences used to amplify a 87-bp fragment of the *Ca*Las rplJ/rplL ribosomal protein gene

Primer	Sequence (5’ to 3’)
CQUL-F	TGGAGGTGTAAAAGTTGCCAAA
CQUL-R	CCAACGAAAAGATCAGATATTCCTCTA
CQUL-Probe	ATCGTCTCGTCAAGATTGCTATCCGTGATACTAG
