# Supplementary material for: Utilization of somatic fusion techniques for the development of HLB tolerant breeding resources employing the Australian finger lime (Citrus australasica)
Source: PLoS One. 2021 Aug 10;16(8):e0255842. doi: 10.1371/journal.pone.0255842 (PMC8354479; doi:10.1371/journal.pone.0255842)
Supplement: S4 Table — (PDF) [file pone.0255842.s005.pdf]

S4 Table. List of the primer sequences used in SYBR Green based real-time PCR assay.

Description (gene symbol)	GenBank accession number	Forward and reverse primer sequences (5' to 3')
PR1	XM_006486769.3	AACTCGCCTCAAGACTACCT<br>CTGCAACTGTGTCGTTCCATA
PR2	XM_015534320.2	ACTTCGCTCAGTACCTTGTTC<br>GGCAGTGGAAACCTTGATTTG
2-oxoglutarate and Fe dependent oxygenase superfamily protein (2OG-Fe)	XM_006490040.2	GGACTTGACGAACGTTGCTT<br>ATCAAGCAGCATGAAGCTTTG
auxin-responsive family protein (ABF3)	XM_006487050.3	AGCGGTGCTCTGGAGAAAG<br>CTCTGCCTGTTTCCTCTCCAA
zinc transporter 10 precursor (ZIP10)	XM_006481314.3	CTATCGGGACTCTGATGATT<br>CCCAACTTTCTCTTCATCTG
calcium dependent calmodulin (CAM8)	XM_006489415.3	TGACGCTGAGGAAGAGCTTAAA<br>ATCACCGTCCAAATCAGCCTC
Expansin (EXP-A4)	XM_006486407.3	CCTGTGGGCATCATTTGTATT<br>AACGCCGTATCCTTGACTGT
ABC transporter C	XM_006483973.3	AGCATCTATCGATACAGCAA<br>CAAGAACAAGAACCAAGTCA
β-actin	XM_006464503.3	GCTGCCTGATGGCCAGATC<br>AGTTGTAGGTAGTCTCATGAA
